# Supplementary material for: Cancer patient survival can be parametrized to improve trial precision and reveal time-dependent therapeutic effects
Source: Nat Commun. 2022 Feb 15;13:873. doi: 10.1038/s41467-022-28410-9 (PMC8847344; doi:10.1038/s41467-022-28410-9)
Supplement: Supplementary file 5 — Supplementary Dataset 2 [file 41467_2022_28410_MOESM5_ESM.zip › Supplementary Data File 2/ICI- Fig 3, S4/Scan time correlation.pdf]

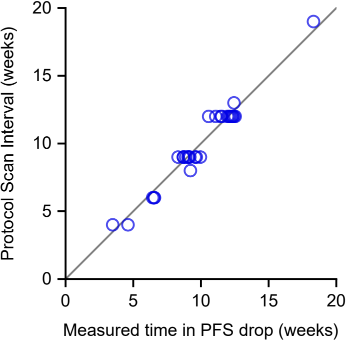

Protocol Scan Interval (weeks)
Measured time in PFS drop (weeks)
0
5
10
15
20
0
5
10
15
20
